# Enriching traditional didactic teaching in undergraduate ophthalmology with lateral thinking method: a prospective study

**DOI:** 10.1186/s12909-022-03443-2

**Published:** 2022-05-17

**Authors:** Mohd-Asyraaf Abdul-Kadir, Lik Thai Lim

**Affiliations:** 1 Department of Ophthalmology, Universiti Malaysia Sarawak (UNIMAS), Kota Samarahan, Malaysia; 2 Department of Ophthalmology, Kulliyyah of Medicine, International Islamic University Malaysia (IIUM), Kuantan, Malaysia

**Keywords:** Medical education, Lateral thinking pedagogy, Ophthalmology, Undergraduate teaching

## Abstract

**Purpose:**

To evaluate undergraduate medical students’ perspective on lateral thinking pedagogy in teaching clinical ocular anatomy in correlation to developing differential diagnoses and recognising red flags in managing common eye conditions.

**Methods:**

The prospective study compared the lateral thinking method (LTM) versus the traditional didactic method in teaching clinical ocular anatomy. Two hundred seventy-two medical students who underwent ophthalmology clerkships were recruited over 3 years. They were randomised into two groups, the LTM and regular didactic groups. Students participated in pre and post-tests to assess their theoretical clinical ophthalmic knowledge, and their responses to respective teaching methods were measured via the self-assessment questionnaire (SAQ), which incorporated a five-point Likert-type scale.

**Results:**

Overall, the LTM group scored significantly higher than the control group, and they found the innovative teaching method improved their confidence and awareness in theoretical knowledge in generating differential diagnoses, managing common eye conditions and recognising potential signs that could be sight and/or life-threatening compared to the regular teaching group (*P* < 0.05). However, all students were neutral towards both lectures regarding changing their negative perception of the current ophthalmic curriculum.

**Conclusion:**

From the student’s perspective, LTM is an effective tool in enriching regular teaching. The method encouraged versatile thinking patterns while enhancing the effectiveness of learning experience in time and resource-limited undergraduate ophthalmic teaching.

## Introduction

The ophthalmic undergraduate curriculum has been criticised for its marginalisation and absence, as limitations in time and resources are rising to accommodate other non-ophthalmic curricula [1–3]. Studies among the primary care and junior doctors have shown their undergraduate ophthalmic teaching was inadequate while they were expected to deliver first-line eye care as ophthalmic presentations could make up to 19% of the primary care visits [4–6]. Moreover, the lower confidence and poorer understanding of ophthalmic knowledge had led to over-referral of benign eye cases, failure to screen and mismanage potentially sight-threatening eye conditions [7, 8]. The rapidly growing prevalence and financial burden of visual impairment in parallel with the increasing ageing population will require all physicians to be better equipped with ophthalmic skills [9].

Simply adding more time for the standalone ophthalmology rotation is inadequate, as Albert and Bartley argued that integrating ophthalmology teaching with other specialities like primary care or internal medicine would be more fruitful [10]. Moreover, traditional didactic teaching in an ophthalmology curriculum is not enough as transfer of skills is also warranted. Thus, there is a growing niche to enrich teaching ophthalmic curricula for medical students, and these include simulation training using an ophthalmoscope [11] and virtual ophthalmic surgery [12], smartphone fundoscopy [13], fundus photography [14], competency-based curricula [15, 16], flipped classroom [17, 18] and team-based learning [19].

Therefore, the focus has shifted in emphasising productivity of the time-limited students. We introduced lateral thinking method (LTM) to enhance traditional didactic teaching, which focuses on the lateral thinking method to improve students’ long-term understanding, comprehension and appreciation of ophthalmic knowledge and equally important the effective practical application for the safe management of patients while nurturing students’ interest in ophthalmology. The framework of the LTM was developed by one of the authors, LTL which was based on his clinical teaching experience in the United Kingdom and Malaysia. He addressed the major challenges specifically in providing undergraduate ophthalmic education in both countries, particularly the lack of interest and ability to apply the right ophthalmic knowledge as expected at their level despite the limited clinical exposure [20]. LTL’s cumulative experience in delivering ophthalmic education motivated the development of LTM in bridging these gaps in undergraduate ophthalmology.

Edward de Bono propagated the lateral thinking method, a dimension of creative thinking to reconstruct and transform information into new ideas regardless of order and sequence [21]. It also highlights creativity to encourage learners to apply prior knowledge more productively, thus expanding the knowledge breadth [22]. This prospective study aimed to enhance the current trend in teaching the ophthalmology curriculum and create active awareness of LTM in medicine through these transferable skills.

## Methods

### Study population

Two hundred seventy-two students were recruited from October 2016 to October 2019. They were Fourth Year Medical Students from Universiti Malaysia Sarawak (UNIMAS) who underwent a 3-week ophthalmology clerkship as part of the Doctor of Medicine (MD) program. The cohort was then randomly assigned by using the online randomizer tool, “https://www.randomizer.org” into two groups, namely LTM (*n* = 136) and Control or traditional didactic (*n* = 136) groups. The study was approved by the institutional ethics review board of UNIMAS, written informed consent was obtained from each participant and the study was conducted in accordance with the Declaration of Helsinki.

### Study design

The LTM and the control groups were taught by senior ophthalmologists with at least 5 years of clinical teaching experience. LTM is a generic systematic didactic teaching approach incorporating three additional components to enhance the value and retention of the teaching effects through:

- Appreciating presenting symptoms and linking it with the possible anatomy of the eye and based on that to take a targeted history.
- In all cases, to think along the lines of life and/or sight-threatening conditions.
- To think laterally and associate with other medical and surgical specialities based on a) and b) to generate meaningful differential diagnoses and effective management plans.

**Fig. 1:**
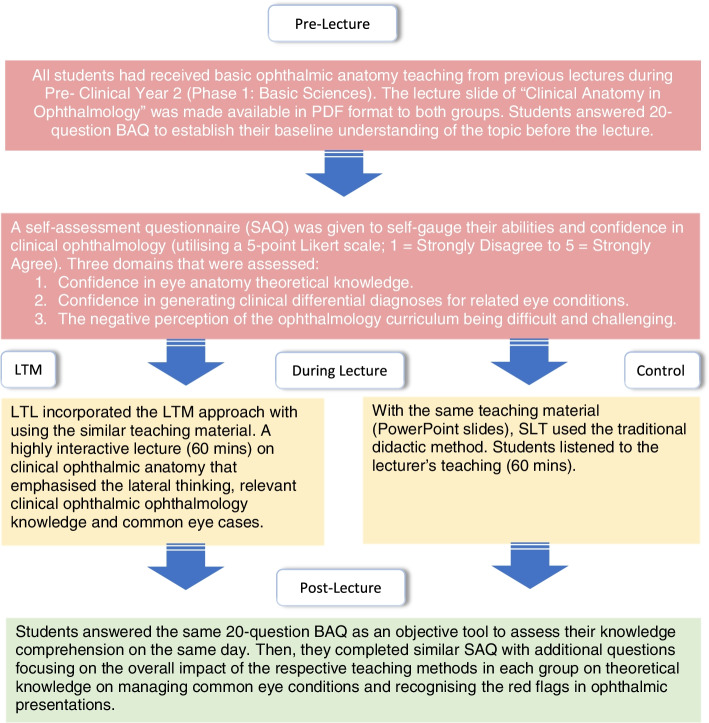
Summary of methodology

**Fig. 2:**
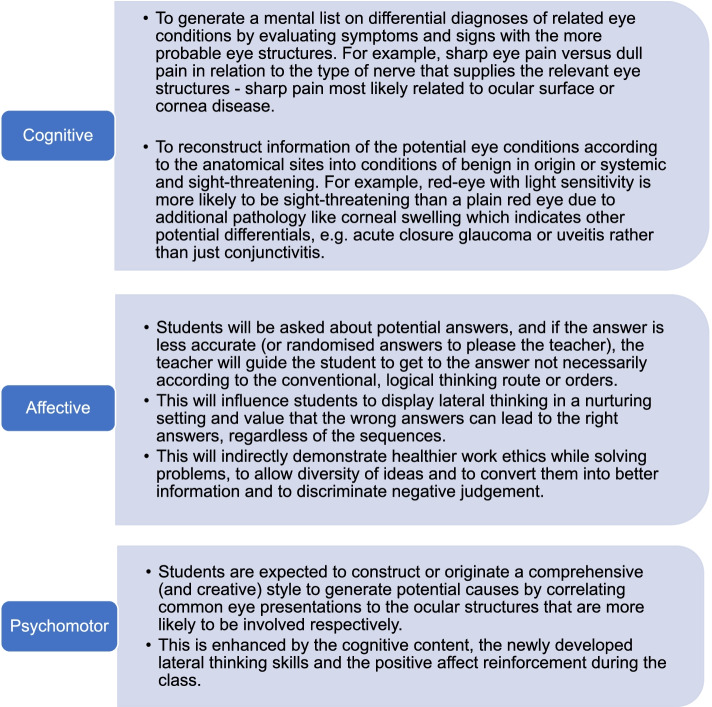
Adaptation from Bloom’s Taxonomy Learning Domains

Teaching ophthalmic anatomy can be redundant and dull as it heavily relies on passively memorising a copious number of facts and names which can be disengaging to students. With LTM, teaching eye anatomy and laterally associating the ophthalmic knowledge with other bodily systems can maximise the impact of learning within a limited timeframe under the guidance of an experienced clinician to ensure fundamental clinical pearls are not missed. At the same time, the LTM method explores the width and breadth of eye presentations and their causative risk factors according to their potential anatomical sites to generate meaningful lists of differential diagnoses. In addition, it only requires a single education provider instead of recruiting few tutors to conduct case-based learning groups given the limited funding on teachers and resources. Figures 1 and 2 summarised the study methodology and the components addressed in LTM according to the Bloom’s taxonomy domains [23].

### Statistical analysis

The pre and post-test scores were analysed between the two groups by an independent samples t-test. The questionnaire data were analysed using the Mann-Whitney-U test. All statistical analyses were performed in the SPSS statistical package version 26.0 (SPSS Inc., Chicago, IL, USA). All data were reported in means and standard deviations (SD) and a *P*-value of < 0.05 was considered statistically significant.

## Results

**Fig. 3:**
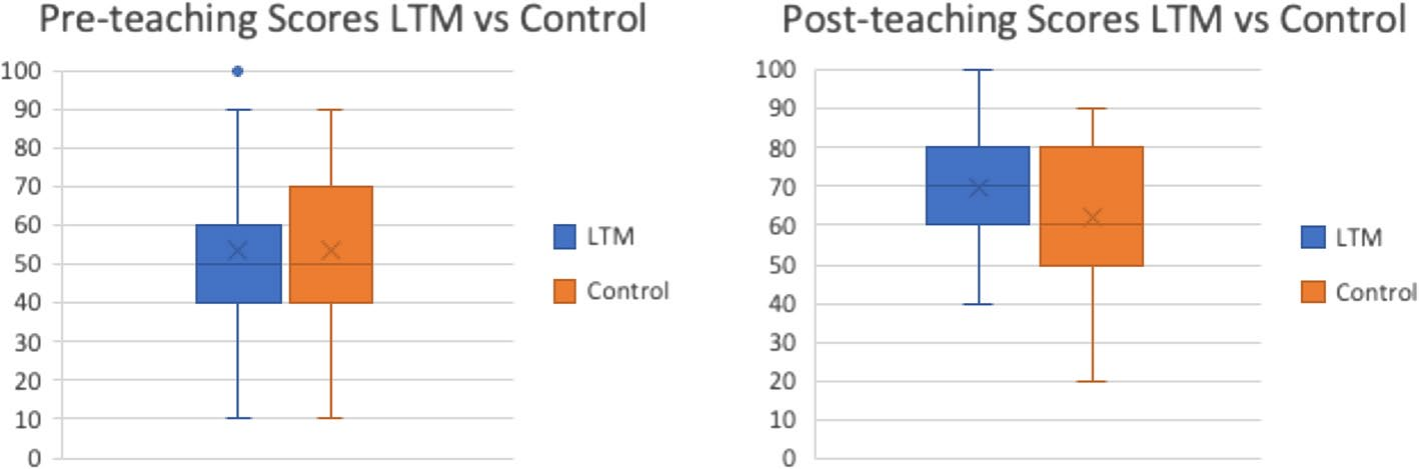
Demonstrated scores distribution between two groups, LTM vs Control in pre and post teaching

A total of 272 students were recruited in the study and participated in both tests, pre and post intervention. Reliable and completed SAQ response rates were 88% (*n* = 120) in the LTM group and 90.4% (*n* = 123) in the Control group. Both LTM and control groups showed comparable knowledge prior to teaching with mean score of 53.53 + 15.8 vs 53.53 + 16.49, t = 1 (df = 135) and *P* = 0.31. Post-test demonstrated the LTM group achieved significantly higher scores than the traditional teaching group with mean score 69.85 + 14.0 vs. 61.76 + 17.2, t = < 0.00 (df = 135) and *P* = 0.01. The scores distributions were depicted in Fig. 3 box and whisker plot.

The questionnaire data in Table 1 demonstrated that before and after the lectures, students in both groups agreed that their eye anatomy knowledge was comparable. All the students also reported that they were neutral to both lectures to have an impact on alleviating the ophthalmic curriculum’s negative perception. Interestingly, the control group was more confident in generating differential diagnoses for eye conditions before the anatomy lecture than the LTM group (Z = − 2.526, *p* = 0.012). However, post-lecture, the LTM group demonstrated a statistically significant difference in their confidence in synthesising differential diagnoses than the respective control group (Z = -3.044, *p* = 0.002). Overall, the LTM group felt the innovative pedagogy had improved their knowledge and confidence significantly to manage common eye disorders (Z = − 3.123, p = 0.002) as well as recognising potential red flags in eye-related conditions (Z = − 2.339, *p* = 0.19) in comparison to their peers in the control group.

**Table 1:** Comparison of students’ responses between LTM and Control groups; before and after lecture and overall responses

Questions	LTM, *n* = 120		Control, *n* = 123		*P* value^a^	Effect size (r)^b^
	Median	Mean + SD	Median	Mean + SD		
Before lecture						
Knowledge of ocular anatomy is good **before** the anatomy lecture	3	2.72 + 1.14	3	2.94 + 1.04	0.103	0.10
Good confidence in making differential diagnoses **before** the anatomy lecture	2	2.05 + 0.88	2	2.32 + 0.88	0.012*	0.16
I perceive the undergraduate ophthalmology course **before** the lecture to be difficult and challenging	3	3.41 + 0.92	3	3.34 + 0.82	0.634	0.03
After lecture						
Knowledge of ocular anatomy is good **after** the anatomy lecture	4	4.04 + 0.56	4	3.86 + 0.74	0.074	0.11
Good confidence in making differential diagnosis **after** the anatomy lecture	4	3.71 + 0.70	3	3.38 + 0.83	0.002*	0.20
I perceive the undergraduate ophthalmology course **after** the lecture to be difficult and challenging	3	3.05 + 0.92	3	3.16 + 0.90	0.396	0.05
Overall						
The anatomy lecture has been useful to my management of common eye conditions.	4	4.06 + 0.63	4	3.78 + 0.73	0.002*	0.20
The anatomy lecture has been useful to me in identifying potential sight and/or life-threatening eye-related conditions.	4	4.18 + 0.69	4	3.98 + 0.74	0.019*	0.15

^a^ = Two groups are compared by Mann-Whitney-U test and p < 0.05 is considered statistically significant

^b^ = Effect size (r) is determined by divided by the root of sample size (small effect: 0.1 < *r* ≤ 0.3, medium effect: 0.3 < *r* ≤ 0.5, large effect: *r* > 0.5)

Likert Scale; 1 = Strongly Disagree, 2 = Disagree, 3 = Neither agree nor disagree, 4 = Agree, 5 = Strongly Agree

## Discussion

The study assessed the impact of lateral thinking pedagogy in teaching undergraduate ophthalmology from the students’ perspective. Lateral thinking teaching method has been implemented and studied in more analytical courses like engineering [24, 25]. Frequently, educators encouraged their students to think “laterally” without labelling it, yet further instrumentation of this pedagogy has not been studied in undergraduate medical teaching. Lateral thinking is seen as a tool to reform thinking patterns, to nurture new ideas, focuses on the process and less on the final results whilst order and sequence do not matter [21]. It is complementary to the vertical thinking method, a hierarchical process that establishes every step has to be correct before moving to the subsequent stage [26].

Overall, the LTM group found the method improved their confidence in ophthalmic clinical knowledge, managing common eye conditions and recognising signs of visual or life-threatening eye diseases. We found the LTM stimulates students’ interest as the provider’s direct instruction or question during the lecture engages and challenges students’ thinking; to encourage them to think of novel ideas about the given topic. By not stressing the order of the thinking, the students can work out their thinking process laterally or even backwards. Furthermore, by not sticking to a routine or rigid structure of processing knowledge or inputs, students could expand their knowledge’s breadth (and depth) and make associations of any “invisible” matters to the information given. This form of active learning could improve students’ long term memory compared to the regular lecture classroom, which can be affected by medical students’ short attention span [27]. Moreover, the method also encouraged higher-order of learning processes in the learning domains as illustrated in Table 1.

The advantage of this method compared to the flipped classroom, which had gained much popularity in teaching ophthalmology undergraduate [17, 18, 28, 29] and postgraduate [30] level is that the students did not have to commit to any specific pre-requisites readings or recorded lectures before the teaching. The LTM’s goal is for students to make meaningful associations of the current learning material to their previously learned knowledge that was not necessarily confined to the field of ophthalmology. Since the ophthalmology curriculum has been made increasingly distant and more peripheral from other clinical specialities, students may find initiating these connections difficult and complex. A teaching tool like LTM can accommodate these gaps in the undergraduate ophthalmology teaching.

In addition, the questionnaire data revealed that the students had a neutral response with no preference towards either teaching methods to alleviate their anxiety or negative preconception that the undergraduate ophthalmology curriculum was challenging. This finding is not surprising given students’ short, allocated time to explore ophthalmology with limited exposure to the clinical setting [20, 31].

We identified that the study was lacking of longitudinal follow-up as we assessed the students’ responses once-off after the lectures. A crossover design would be ideal to compare strengths and disadvantages of each teaching method however the short-allocated time for the ophthalmology clerkship of the entire clinical phase of the MD programme makes such implementation less practical. The pedagogy can be further studied on the impact of students’ long-term memory and retention via reassessment after some time without a prior revision. While flipped classroom promotes online and digitalisation of education with the pre-recorded lectures, lateral thinking pedagogy heavily depends on the teachers to actively deliver efficient teaching materials to allow active engagement and a safe place to generate mistakes and learn from them. Therefore, training the providers can be practical and effective especially if they are familiar with the creative or lateral thinking methods which may be cost and resource-effective in delivering undergraduate ophthalmic teaching as demonstrated by the students’ scores.

Re-evaluation and reformation of the questionnaires to address other areas of students’ perspectives could be imposed in future studies, including their enthusiasm for pursuing ophthalmology and their confidence in solving unfamiliar problems. Furthermore, the study was limited to selected key topics associated with clinical ophthalmic anatomy, which relies primarily on remembering older information rather than reforming new ideas and solutions that could encourage creativity. The topic that is too structured and less abstract may not be suitable for the lateral thinking pedagogy, although it relatively emphasises the long-term memory formation and restructures the knowledge into a purposeful clinical application.

Moreover, the study was culturally challenging as the students who have been exposed to long-standing vertical and analytical thinking may find it challenging to express creative thinking skills publicly. As lateral thinking enforces finding solutions regardless of the orders and sequences, students may find expressing “wrong” answers can be humiliating in front of their peers. They were used to be rewarded for giving correct, valid answers rather than original, unusual ideas and empowering resilience and celebrating creative learners can be challenging. The lateral thinking teaching techniques may vary among the students’ cohorts as the provider taught more, the better he was at the proposed pedagogy and thus benefit the newer groups more than the older ones.

As the undergraduate medical curriculum becomes more complex and arduous, ophthalmic education time has lessened, with more resources given to other specialities and the diminished role of primary care physicians in community eye care [32]. Therefore, the LTM approach emphasising patient’s safety, systematic lateral analytical thinking and the practical application of knowledge can be an impactful approach to teaching undergraduate ophthalmology. Furthermore, such an approach can be applied to other fields of medicine, thus making learning and practising medicine more meaningful for both medical practitioners and patients. By making lateral thinking a habitual teaching practice via structured training and teaching, students can reshape their minds to allow better application of knowledge and versatile ways of thinking within the limited timeframe and resources.

## Data Availability

Data are available upon request to the corresponding author at akmasyraaf@unimas.my.

## Abbreviations

LTM: Lateral Thinking Method
SAQ: Self-assessment questionnaire
BAQ: Best answer question
